# Application of a convolutional neural network for automatic cytological diagnosis of 7 common canine tumors on whole slide images: a proof-of-concept

**DOI:** 10.3389/fvets.2026.1922780

**Published:** 2026-10-13

**Authors:** M. Attini, E. Newing, I. L. Oikonomidis, T. K. Tsouloufi, L. Ressel

**Affiliations:** 1 Independent Practitioner, Leggiuno, Italy; 2 Department of Veterinary Anatomy, Physiology and Pathology, Institute of Infection, Veterinary and Ecological Sciences, Faculty of Health & Life Sciences, University of Liverpool, Liverpool, United Kingdom

**Keywords:** canine, convolutional neural network, cytology, oncology, whole slide image

## Abstract

**Background:**

Artificial intelligence may help improve speed and consistency of diagnostic veterinary cytology, but validated tools remain limited.

**Objectives:**

To develop and evaluate a convolutional neural network (CNN) for automatic classification of 7 common canine tumors on cytology whole-slide images (WSIs).

**Methods:**

Ninety-one May–Grünwald Giemsa–stained canine cytology cases were selected, representing carcinoma, histiocytoma, lymphoma, mast cell tumor, melanoma, plasma cell tumor, and soft tissue sarcoma, together with a background class. Slides were tiled into 22,577 image patches (512 × 512 pixels) and manually labeled and split using a case-wise 3-fold design to avoid data leakage. An ImageNet-pretrained Inception-v3 CNN was fine-tuned for 8-class classification. Performance was assessed at tile level, Grad-CAM was used for qualitative interpretability analysis, and majority-vote prediction was further evaluated at ROI level using attention-guided inference and at WSI level using attention-free whole-slide inference.

**Results:**

Mean test accuracy at tile level was 86.3%, with mean macro balanced accuracy of 92.0% and mean macro F1-score of 85.6%. Grad-CAM showed attention focused predominantly on cytologically relevant cellular regions. At ROI level, attention-guided inference achieved 100% Top1 accuracy (28/28 cases). At WSI level, attention-free whole-slide inference achieved 92.9% Top1 accuracy (26/28 cases), and the correct diagnosis was included within the top 3 predictions in all cases.

**Conclusion:**

Inception-v3 achieved high diagnostic performance on canine cytology WSIs. The results support the feasibility of both AI-assisted ROI-based decision support and fully automated whole-slide inference in veterinary cytology. These findings are proof-of-concept evidence from a curated, single-institution cohort of typical, well-differentiated cases and require validation on diagnostically challenging and external cases before clinical use.

## 1 Introduction

Diagnostic cytology is a cornerstone of clinical decision-making in veterinary oncology, providing a minimally invasive, rapid and relatively inexpensive means of characterizing neoplastic processes. Cytologic diagnosis highly depends on expert interpretation and is prone to inter- and intra-observer variability. In human medicine, these challenges have driven attempts to automate cytologic screening (1).

The widespread adoption of digital slide scanners and WSI formats enables this evolution. Contemporary deep convolutional neural networks (CNNs) have transformed computer vision performance and provide the backbone for most modern medical image analysis systems (2–5). In histopathology, CNNs have been successfully applied to a wide range of diagnostic and prognostic tasks, including tumor detection, grading and biomarker prediction at the WSI level (6). These successes have accelerated interest in applying similar approaches to cytology, where overlapping cells, inherent lack of tissue architecture and staining variability present a natural testbed for representation-learning methods.

In human cytology research, deep learning has already been translated to clinically relevant problems on WSIs. For cervical screening, Cheng and colleagues proposed a multi-stage system that combines low- and high-resolution ResNet-based models and a recurrent neural network for slide-level triage on more than 3,500 liquid-based cytology WSIs, achieving sensitivities and specificities comparable to expert cytopathologists while substantially reducing review workload (7). Another group trained a WSI-level model to classify cervical liquid-based cytology specimens as neoplastic versus non-neoplastic, reaching AUCs of 0.89 to 0.96 across independent test sets demonstrating the feasibility of robust, fully automated triage using digitized WSIs (8).

Beyond cervical screening, CNNs have been applied to other cytology specimen types: Tsuneki and colleagues developed a system for urothelial carcinoma screening in urine cytology WSIs, reporting high diagnostic performance and suggesting that AI-assisted urine cytology could standardize the application of the Paris System and reduce false-negative rates (9). More broadly, commercial and research-grade systems are emerging for blood smears, bone marrow, effusions and fine-needle aspirates, with AI models assisting in differential counts, blast detection and malignancy triages (1, 10). These studies suggest that CNN-based analysis of cytology WSIs can achieve clinically useful performance, integrating into existing laboratory workflows.

In veterinary medicine, cytology is arguably more central to diagnostics than in human medicine, as it enables rapid, low-cost assessment of solid organs/lesions and body cavity effusions in first-opinion practice (11, 12), but while histologic applications are comparatively well developed, there remains a relative paucity of validated AI tools for veterinary cytology (13).

Recent reviews on digital veterinary clinical pathology document the rapid growth of cytology platforms, including point-of-care scanners and teleconsultation services, and highlight the emerging potential of AI-driven image analysis for blood, bone marrow and cytology preparations, while emphasizing the need for quality control and decision support, and underscore practical challenges such as data curation, ground-truth definition and ethical considerations (14, 15).

One of the few fully developed examples is the work by Marzahl et al., who used deep learning to quantify pulmonary hemosiderophages in equine bronchoalveolar lavage cytology slides for assessing exercise-induced pulmonary hemorrhage (16). Their CNN-based approach outperformed conventional semi-quantitative scoring, but it targeted a narrow, single-task endpoint rather than broad diagnostic categories (13, 14, 16).

To our knowledge, no published work has yet evaluated a multitask CNN trained on WSIs from a diverse set of small-animal tumor lesions. Building on the solid Inception-v3 architecture in large-scale image classification and medical imaging tasks (5), we developed and evaluated this CNN for automated classification of veterinary cytology WSIs into these seven diagnostically relevant classes. Our aim was to assess whether such a model could achieve relevant performance at tile and WSI levels, thereby providing support for future AI-augmented veterinary diagnostic cytology workflows.

## 2 Materials and methods

### 2.1 Case selection and digitalisation

Cases were selected from University of Liverpool cytology archive by one Author (IO) aiming at typical morphologically diagnostic cases according to established cytomorphological criteria described in the literature (17–19), excluding poorly differentiated tumors that would otherwise require histology and/or immunohistochemistry for the final diagnosis. Tumor classes were: mast cell tumor (MCT), lymphoma (LYM), carcinoma (CAR), soft tissue sarcoma (STS), melanoma (MEL), histiocytoma (HIS), and plasma cell tumor (PCT). These were reviewed by two board-certified pathologists (LR, TT); cases with concordant diagnosis were selected for inclusion in the population, while those considered of general poor quality were discarded. For each selected case, an MGG-stained slide was digitized as a whole-slide image (WSI) in RGB color format using a digital slide scanner (Aperio CS2, Leica Biosystems, Wetzlar, Germany) at 400x final magnification as an. SVS file.

### 2.2 Image pre-processing

Across the WSI, a smaller region of interest (ROI) centered on the most representative/cellular area of the slide was manually selected by the operator using Aperio Imagescope software (Aperio, Leica Biosystems), and a new. SVS file was obtained. To generate a tile-level image dataset from the ROIs, we implemented a custom tiling pipeline in MATLAB (MathWorks, Natick, MA, USA) using the Bio-Formats library (bfGetReader, bfGetPlane, bfopen; Open Microscopy Environment). For each slide, a Bio-Formats reader was initialized, and all image series were queried at the highest-resolution plane (400× final resolution) and the tile size set at 512×512 with no overlap. From these inputs, the script computed the vertical and horizontal step size between tile origins, and the slide was scanned in a regular grid. For each tile position, the corresponding ROI was read from the selected series and tiles were saved as. JPG files. After tiling was completed for a given WSI, a post-processing step removed any non-square tiles arising from incomplete tiles at image borders. This procedure was applied sequentially to all WSIs and resulting images (tiles) constituted the raw image dataset. A visual summary of the process is depicted in Figures 1E–H.

**Figure 1:**
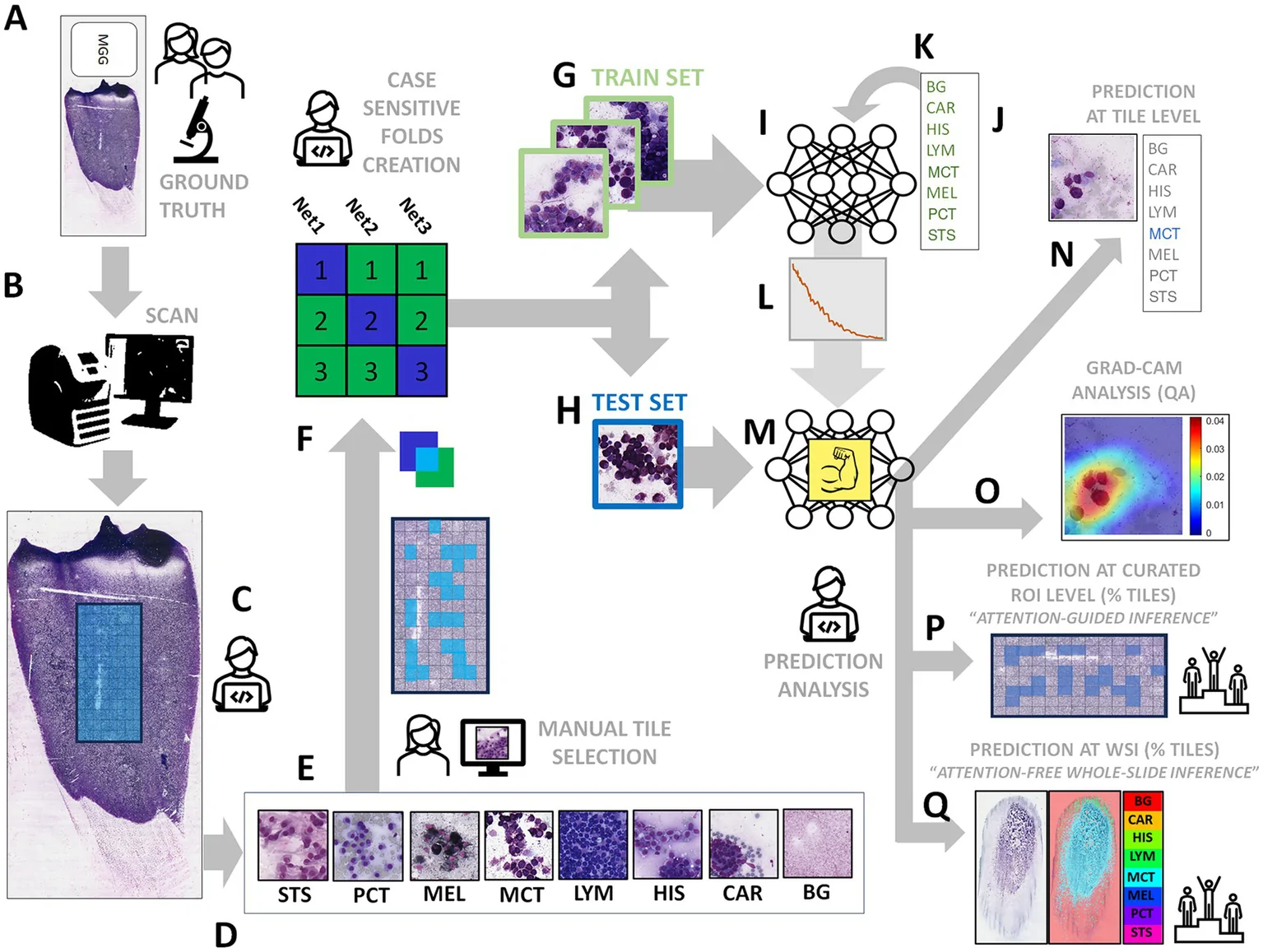
Workflow used for development, training, testing, and whole-slide application of the convolutional neural network (CNN) for canine cytology whole-slide images (WSIs). **(A)** Cytology slides stained with May–Grünwald Giemsa (MGG) were reviewed. **(B)** Selected slides were digitized as WSIs at 400 × magnification. **(C)** A representative region of interest (ROI) was manually selected from each WSI. **(D)** The selected ROI was automatically tiled using a custom MATLAB/Bio-Formats pipeline into non-overlapping 512 × 512 pixel image patches. **(E)** Tiles were manually reviewed and selected for class labeling as sarcoma (STS), plasma cell tumor/plasmacytoma (PCT), melanoma (MEL), mast cell tumor (MCT), lymphoma (LYM), histiocytoma (HIS), carcinoma (CAR), or background (BG). **(F)** Cases were assigned in a strictly case-wise manner to 3 folds, and fold-specific train/test datasets were generated to avoid data leakage between tiles from the same case. **(G)** Tiles from 2 folds were combined to form the training set. **(H)** Tiles from the remaining fold were used as the independent test set. **(I)** An ImageNet-pretrained Inception-v3 architecture was adapted and trained on the training set. **(J)** The final output layer was configured for 8 classes. **(K)** Training and hyperparameter optimization were performed iteratively via backpropagation. **(L)** Training performance curves were monitored to identify the best-performing model for each fold. **(M)** The selected CNN was then applied to held-out test tiles and to slide-level analysis. **(N)** Tile-level predictions were generated, and **(O)** Grad-CAM was used as a qualitative assessment of the image regions driving classification. **(P)** Tile predictions were aggregated to provide ROI-level class proportions (% tiles) simulating an *attention-guided inference* approach. **(Q)** The trained network was further applied across the slide to reconstruct a spatially resolved classification output showing the original image, the color-coded prediction mask, and the class legend with relative tile proportions, simulating an *attention-free whole slide inference* approach. Human figures without a screen indicate manually performed, human-driven steps, whereas human figures shown with a computer screen and code symbol indicate script- or code-directed computational steps; Podium icons indicate that the final ROI- and WSI-level diagnoses were derived by majority vote. QA, Quality assurance.

### 2.3 Ground truth creation and training/test split

Tiles were manually selected by one operator (MA) according to the following criteria: images representative of microscopic fields where cytologic characteristics were obvious according to published cytologic criteria (17–19) and where diagnostic cellular material represented at least 20% of the tile area. For the background class, tiles were selected containing either: clear, acellular background, proteinaceous background, or blood cells (e.g., erythrocytes, neutrophils, monocytes, etc.) with no tumor-diagnostic cells present. To construct training and test sets while avoiding data leakage between tiles from the same cytology case, we implemented a dedicated MATLAB script (MathWorks, Natick, MA, USA) that operates on the tiled WSI dataset. Although each animal/slide belonged to one tumor class, case slides contributed to non-diagnostic (background class).

### 2.4 CNN training and evaluation at tile level

All convolutional neural networks (CNNs) were trained in MATLAB R2023a (MathWorks, Natick, MA, USA) using the Deep Learning Toolbox, Deep Network Designer app, and Experiment Manager. During training all tiles from a given cytology case remained either in the training or in the held-out test set for that network, but never in both. Within Deep Network Designer, an Inception-v3 architecture pre-trained on ImageNet was imported and the final classification layers replaced by a new fully connected layer and a softmax/classification layer with eight output units, corresponding to the eight categories. During training, all tiles were automatically resized to match this input size (299 × 299 × 3). For each fold, a separate Deep Network Designer project was created (Network 1, Network 2 and Network 3). In each project, the train directory for that fold was loaded as an image datastore, using folder names as categorical labels and enabling random shuffling at the beginning of each epoch. The test directory of the same fold was kept completely separate and was not used during optimization. Fold sizes were reported as class-specific case instances rather than as numbers of unique animals. Each case contributed one instance to its tumor class and, when background tiles were available, one additional instance to the background class. Thus, the total number of class-specific instances was calculated as the sum of the tumor-class instances and background instances. For example, the Network 3 training set contained 9 cases in each of the 7 tumor classes, corresponding to 63 tumor-class instances (7 × 9), plus 60 background instances, yielding 123 class-specific case instances. Hyperparameter optimization was performed using Experiment Manager. For each fold, we defined experiments that systematically compared the main solvers provided in the app (“rmsprop,” “sgdm,” “adam”) across three initial learning rates (0.1, 0.01 and 0.001). Each combination of solver and learning rate was run as an individual trial, using otherwise identical settings (batch size, number of epochs, data augmentation, and L2-regularization). Training and test performance curves (accuracy, loss) were monitored within Experiment Manager, and networks were trained for different numbers of epochs (5,10,15), choosing the best result to avoid overfitting. The best configuration for each fold was selected based on test set performance and convergence behavior (highest test accuracy with stable loss and no evidence of progressive overfitting). The final networks reported in this study (Network 1–3) correspond to the best-performing solver/learning-rate/epochs combinations for each. Confusion matrices were obtained for both training and test sets. Once training for a given fold was completed and the optimal configuration identified, the resulting network was exported from Deep Network Designer to the MATLAB workspace and saved as a .mat file. These three trained networks (representative of each fold split) were then used programmatically for tile-level evaluation on their respective held-out test sets. For all trials, the mini-batch size was 128, and the L2-regularization coefficient was 0.0001. Data augmentation comprised random reflection about both the *X* and *Y* axes, random rotation from 0 to 30 degrees, and random horizontal and vertical translation from 0 to 0.3 pixels. The random rescaling range was 1 to 1 and therefore did not introduce scale augmentation. Training was performed on a Windows 11, A9 CPU, 64GB RAM, 1xNvidia RTX6000 workstation. A visual summary of the process is depicted in Figures 1I–N.

### 2.5 Grad-CAM analysis of tiles

To visualize the image regions that contributed the most to the CNN predictions on the test set, we implemented a batch Grad-CAM pipeline in MATLAB (MathWorks, Natick, MA, USA), using a custom script. The network with average test accuracy among Networks 1–3 was selected to test Grad-CAM analysis. To identify the feature maps used for Grad-CAM computation, the script automatically detected the last convolutional layer in the network ensuring that class-discriminative localization maps were computed from the deepest convolutional representation available. The script recursively enumerated all files within a root directory and for each image, spatial resizing to match the network’s input height and width, and adjustment of the number of channels to match the network input were performed. Each pre-processed image was classified using the network, and the predicted label mapped to the corresponding class index from the classification layer. This predicted class index was used as the target class for Grad-CAM. For every image, a Grad-CAM heatmap was computed. As output, for each processed image, a figure was generated containing both the resized original image, and the same image overlaid with the Grad-CAM heatmap with 50% transparency and an accompanying color bar. Grad-CAM output was evaluated qualitatively on a randomly selected population of tiles (100 tiles per category in the test set). A visual summary of the process is depicted in Figure 1O.

### 2.6 Analysis of CNN prediction at ROI level with majority vote (attention-guided inference)

To extend CNN predictions from the individual tile level to the sum of the tiles from the region-of-interest (ROI) level of the test cases, simulating a human-guided diagnostic workflow in which the CNN acts as a decision-support copilot, we implemented a custom MATLAB script that classified all tiles belonging to the same ROI of a single case and summarized the distribution of predicted classes by majority vote. The network with average test accuracy among Networks 1–3 was selected for this analysis. After all tiles were processed, the script generated a summary for each ROI, reporting the total number of classified tiles, the number of tiles assigned to each category, and the corresponding percentage of the ROI represented by each predicted class. ROI-level interpretation was then derived by majority vote, whereby the class with the highest number of predicted tiles was considered the final ROI-level classification. A visual summary of the process is depicted in Figure 1P.

### 2.7 Analysis of CNN prediction at WSI level with majority vote (attention-free whole slide inference)

To assess performance at the level at which cytology is actually reported (the whole slide) simulating a fully automated AI approach, we applied the CNN to the whole WSIs from which test tiles originated from and derived a single diagnosis per slide by majority vote across non-background tiles. This extended predictions to the entire slide area, not necessarily diagnostic and not seen during training or testing, simulating a fully automated screening workflow, in which the algorithm must both encounter diagnostically relevant and non-relevant areas autonomously and generate a slide-level prediction without human direction. For each WSI, all tiles predicted as background were excluded and the remaining tiles were grouped by predicted tumor classes. The class accounting for the highest percentage of non-background tiles (Top1) was taken as the slide-level prediction; the second and third most frequent classes (Top2 and Top3) were also recorded. As an additional descriptive analysis of ROI-level and WSI-level class separation, the Top1–Top2 margin was calculated as the difference between the tile percentages assigned to the highest-ranked and second-highest-ranked classes. Margins were expressed in percentage points and summarized using the mean, median, and range. The numbers and percentages of ROIs and whole slides with margins below 5 and 15 percentage points were also reported. These thresholds were descriptive and did not alter the Top1 classification rule. The network with average test accuracy among Networks 1–3 was selected for WSI-level analysis: Whole-slide images (WSIs) of cytology smears from test cases were processed in batch using a custom MATLAB script (MathWorks, Natick, MA, USA) built around the Bio-Formats library. The script scanned a user-defined input directory, identified all supported WSI files, and processed them sequentially. In each position, a tile was extracted using the appropriate ROI coordinates and all tiles were classified using the CNN. The predicted class label was mapped to its index in the network’s final layer, and class-wise tile counts and tile pixel areas were accumulated for the entire slide. In parallel, a color-coded visualization tile was generated for each image: a solid RGB mask color was assigned to each class based on a color map [background (BG): red; carcinoma (CAR): amber/golden yellow; histiocytoma (HIS): lime green; lymphoma (LYM): spring green; mast cell tumor (MCT): cyan/aqua; melanoma (MEL): royal blue; plasma cell tumor (PCT): violet; and soft tissue sarcoma (STS): magenta/fuchsia] and blended with the original tile at 40% opacity to preserve underlying morphology. To provide a compact slide-level visual summary, a strongly downsampled (5% of the original WSI dimensions in each axis) composite. Jpg image was reconstructed from the classified tiles. For quantitative reporting, a plain-text file was produced for each slide. This report listed, for every class, the number of tiles assigned to that class, the total number of pixels covered by those tiles, and the percentage of the slide area they represented relative to the sum of all classified tile pixels. The original WSI dimensions and requested base tile size were also recorded. A visual summary of the process is depicted in Figure 1Q.

### 2.8 Statistical analysis

Statistical analysis was descriptive. For each fold, performance at tile level was assessed on both training and independent test sets using confusion matrices and standard multiclass classification metrics, including overall accuracy, macro balanced accuracy, macro F1-score, weighted F1-score, Cohen’s kappa, and multiclass Matthews correlation coefficient (MCC). Class-specific performance was described using precision, recall, and F1-scores.

#### 2.8.1 Ethics statement

The project was approved by the Ethics Committee of the School of Veterinary Science, Liverpool, Reference ID: VREC1697.

## 3 Results

### 3.1 Dataset composition and case-wise cross-validation splits

The final cytology dataset comprised 91 (13 per class) independent cases, corresponding to 7 diagnostic categories: carcinoma (CAR), histiocytoma (HIS), lymphoma (LYM), mast cell tumor (MCT), melanoma (MEL), plasma cell tumor (PCT), and soft tissue sarcoma (STS). Background class (BG) originated from non-diagnostic areas of 89/91 cases. This yielded 22,577 image patches (tiles) overall: 4,025 BG tiles and 18,552 tumor tiles. For the neoplastic categories, almost all cases contributed 204 tiles each (204 × 13 = 2,652 tiles per class), except for a single melanoma case that contained 192 tiles (total 2,640 melanoma tiles). The full dataset is available at Zenodo.org (DOI: 10.5281/zenodo.19493409).

Across the three case-wise folds, training and test partitions comprised 56–63 and 28–35 unique cases (animals), respectively, corresponding to 115–123 and 57–65 class-specific case instances and 14,215–15,517 and 7,060–8,362 tiles. Class-specific case-instance totals exceed unique-animal totals because a case could contribute tiles to both its tumor class and the background class, nevertheless, all tiles from a given condition remained within the same training or test partition.

In each fold, the neoplastic classes were approximately balanced at the case level: for every tumor class, 8–9 cases (1,632–1,836 tiles) were assigned to the training set and 4–5 cases (816–1,020 tiles) to the test set. Background was more heterogeneous in terms of tile counts per case but was also split case-wise, with 59–60 BG cases (2,582–2,791 tiles) used for training and 29–30 BG cases (1,234–1,443 tiles) used for testing in each fold.

### 3.2 CNN performance on single tiles

Across the three training folds, the networks showed near-perfect performance: mean training accuracy was 0.9993 (range, 0.9992–0.9994), with mean macro balanced accuracy of 0.9996, macro F1-score of 0.9993, and weighted F1-score of 0.9993. Cohen’s kappa and multiclass Matthews correlation coefficient (MCC) were both 0.9991 on average, indicating almost complete agreement with the reference labels on the training sets.

On the independent test folds, overall performance remained high but, expectedly, showed greater variability between splits. Mean test accuracy across the three networks was 0.8630, with a mean macro balanced accuracy of 0.9201, macro F1-score of 0.8555, and weighted F1-score of 0.8616. The corresponding mean Cohen’s kappa and MCC were 0.8427 and 0.8437, respectively, consistent with strong agreement in a multiclass setting.

At the level of individual folds, test accuracy ranged from 0.793 (Network 2) to 0.901 (Network 1), with Network 3 achieving an intermediate value of 0.895.

Class-wise analysis showed that performance was consistently highest for LYM, MCT, MEL and CAR, and more variable for PCT, HIS, STS and BG. In the best performing fold (Network 1), F1-scores on the test set were ≥0.95 for LYM, MCT and MEL (F1 = 0.975, 0.984 and 0.955, respectively), and approximately 0.89–0.92 for CAR, BG and HIS (F1 = 0.891, 0.918 and 0.884, respectively). In contrast, PCT and STS showed lower F1-scores of 0.756 and 0.831, reflecting a higher rate of misclassification for these categories.

Network 3 showed a broadly similar pattern, with high F1-scores for LYM (0.975), HIS (0.945), CAR (0.909) and PCT (0.884), but reduced performance for MEL (F1 = 0.805), MCT (0.866) and STS (0.810).

The lowest overall test performance (Network 2) was associated with a marked drop in F1-scores for PCT (0.592), HIS (0.651) and STS (0.725), while BG, LYM and MCT remained relatively robust (F1 = 0.938, 0.840 and 0.889, respectively).

Inspection of the confusion matrices revealed that among neoplastic categories, errors were most frequent between PCT, HIS and STS, and between STS and CAR, whereas cross-misclassification between LYM and MCT was rare. Due to the intermediate accuracy of Network 3 (0.895) this was chosen for the following analysis on its test set.

### 3.3 Grad-CAM analysis

Within the test set, BG consistently showed attention focused either on empty background areas, proteinaceous background or blood cells, irrespective of the variation of RBC stain (from red to green). In all the tumor classes, network attention was mainly focused on cellular material and not on non-cellular background areas. In many round cell tumors, attention was clearly focused on individual cells. In tumor types which normally form groups (e.g., epithelial clusters or mesenchymal aggregates), those areas were often used as regions for prediction. Interestingly, in addition to cell groups, in the case of STS, single cell elements were also targeted. Areas characterized by three-dimensional clusters with poor cellular details were not chosen for prediction, while monolayer clusters with better cellular features exhibited high attention values. In round cell tumors, both areas of artifactual cell aggregation/concentration and areas with single cell elements were used for prediction. A summary of Grad-CAM heatmaps from different classes is presented in Figure 2.

**Figure 2:**
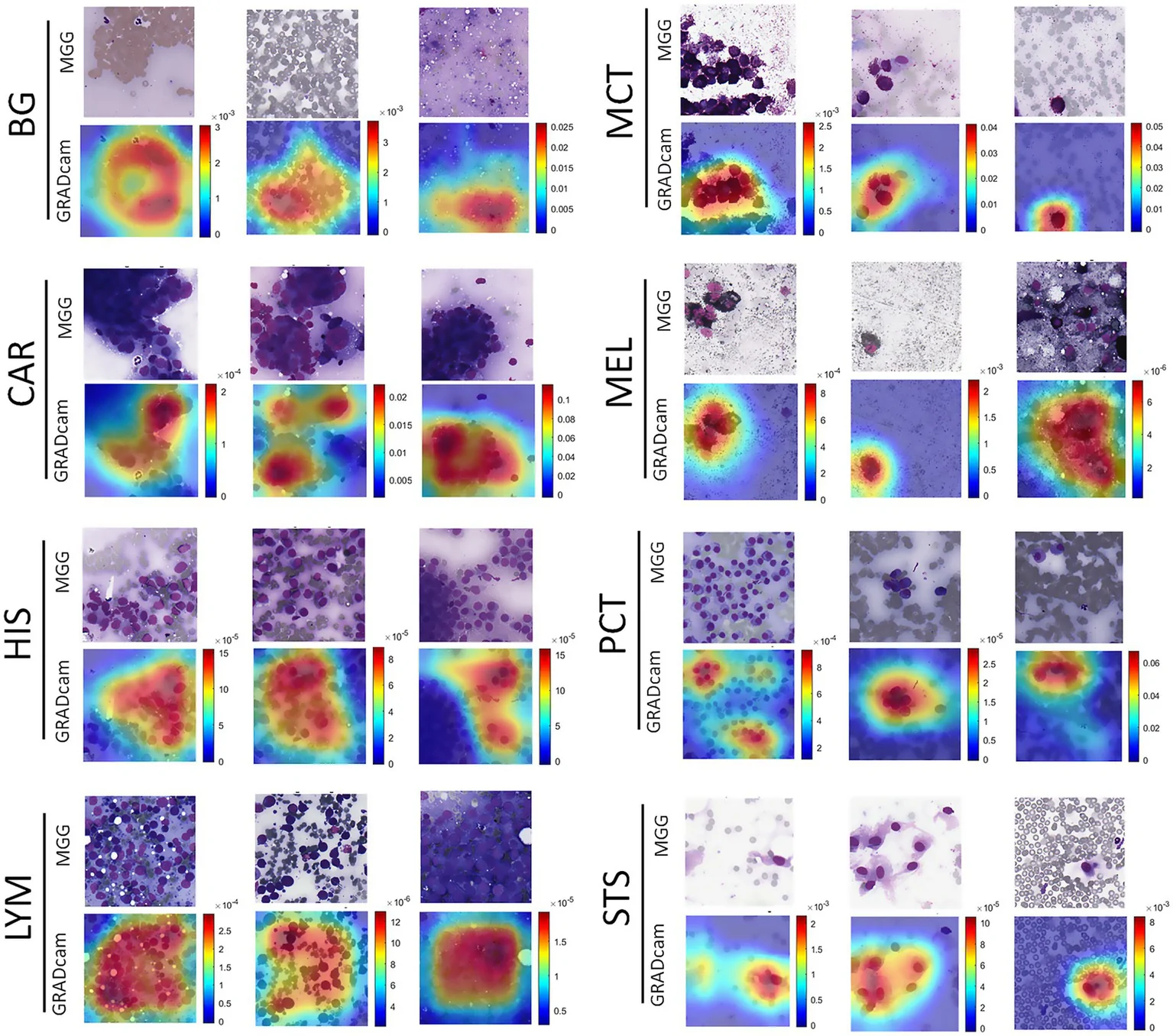
Representative Grad-CAM qualitative assessment of tile-level predictions for the 8 classes. For each class, 3 representative tiles are shown, with the original May–Grünwald Giemsa (MGG) image on the top row and the corresponding Grad-CAM heatmap on the bottom row. Warmer colors indicate the image regions contributing most strongly to the CNN prediction. In general, for neoplastic classes, the network focused predominantly on diagnostically relevant cellular material rather than on non-cellular background. In background (BG) tiles, the main attention was most commonly directed to clear background, acellular/proteinaceous areas and erythrocyte-rich regions. In carcinoma (CAR), attention was most often concentrated on cohesive epithelial cell clusters, clearly avoiding three-dimensional structures where cellular detail was suboptimal. In histiocytoma (HIS), the model most commonly highlighted dense aggregates or sheets of round histiocytic cells. In lymphoma (LYM), the main focus was typically on highly cellular populations of monotonous discrete lymphoid cells. In mast cell tumor (MCT), attention was usually centered on mast cell clusters or individual granulated mast cells. In melanoma (MEL), the network most often focused on pigmented melanocytic cells or cell groups containing dense melanin. In plasma cell tumor (PCT), the dominant attention pattern involved plasmacytoid cells, especially areas where eccentric nuclei and perinuclear clearing were evident. In soft tissue sarcoma (STS), the CNN more commonly focused on monolayer regions containing discrete mesenchymal cells or loosely aggregated cells. Color scale bars are shown for each individual heatmap as the dynamic range was generated separately for each image, therefore scale values are not intended for direct quantitative comparison between panels.

### 3.4 ROI test tiles level diagnostic performance (attention-guided inference)

Across all 28 curated ROI test tiles (204 tiles per case), the Top1 prediction matched the true diagnosis in all cases, corresponding to an overall ROI-level accuracy of 100%.

In 21/28 cases, both Top2 and Top3 classes each represented <10% of tiles, consistent with a single dominant diagnosis without substantial differentials. In the remaining 7/28 cases, plausible alternative classes represented >10% of tiles, including 2/4 MCT, 1/4 CAR, 2/4 STS, 1/4 MEL, and 1/4 PCT cases, whereas no such prominent differentials were observed in LYM or HIS. The most heterogeneous examples included 1 STS case in which STS, HIS, and PCT accounted for 31.6%, 30.6%, and 16.3% of tiles, respectively, and 1 MEL case in which MEL and MCT accounted for 50.2 and 48.8% of tiles. At the class level, the median fraction of selected tiles assigned to the correct Top1 diagnosis per ROI ranged from 87.4% for MCT to 100% for MEL, with similarly high median values for LYM (99.8%), HIS (98.3%), and PCT (98.3%), and slightly lower values for CAR (91.4%) and STS (88.4%). Across the 28 ROIs, the mean Top1–Top2 margin was 80.5 percentage points, the median was 95.8, and the range was 1.0–100.0. Two ROIs (2/28; 7.1%) had margins below 5 percentage points: Case 48 (1.0) and Case 73 (1.4). Three ROIs (3/28; 10.7%) had margins below 15 percentage points. A summary of the results is presented in Table 1.

**Table 1:** ROI-level diagnostic predictions obtained by attention-guided inference.

ROI test tiles–level diagnostic performance (*Attention-guided inference*)									
Case	Ground truth	Predicted class (% of tiles)							Final diagnosis
		MCT	LYM	CAR	STS	MEL	HIS	PCT	
4	MCT	**97.5**	0.0	0.0	0.0	2.5	0.0	0.0	MCT
8	MCT	**77.2**	0.0	0.0	0.0	22.3	0.0	0.5	MCT
11	MCT	**100.0**	0.0	0.0	0.0	0.0	0.0	0.0	MCT
14	MCT	**56.9**	0.0	0.0	0.0	43.1	0.0	0.0	MCT
19	LYM	0.0	**99.5**	0.5	0.0	0.0	0.0	0.0	LYM
23	LYM	0.0	**100.0**	0.0	0.0	0.0	0.0	0.0	LYM
26	LYM	0.0	**100.0**	0.0	0.0	0.0	0.0	0.0	LYM
29	LYM	0.0	**95.6**	0.0	0.0	0.0	4.4	0.0	LYM
33	CAR	0.5	1.5	**97.5**	0.5	0.0	0.0	0.0	CAR
37	CAR	0.0	13.2	**77.9**	2.5	0.0	0.0	6.4	CAR
40	CAR	0.0	0.0	**97.1**	0.0	1.0	0.0	2.0	CAR
43	CAR	2.0	4.4	**85.8**	0.0	1.5	0.0	6.4	CAR
48	STS	4.1	3.1	5.1	**31.6**	9.2	30.6	16.3	STS
51	STS	0.0	0.0	0.0	**99.5**	0.0	0.0	0.5	STS
55	STS	0.0	0.0	19.2	**79.8**	0.0	0.0	1.0	STS
59	STS	0.0	0.0	2.0	**97.0**	0.0	0.0	1.0	STS
63	MEL	0.0	0.0	0.0	0.0	**100.0**	0.0	0.0	MEL
66	MEL	0.0	0.0	0.0	0.0	**100.0**	0.0	0.0	MEL
70	MEL	0.0	0.0	0.0	0.0	**100.0**	0.0	0.0	MEL
73	MEL	48.8	0.0	0.0	1.0	**50.2**	0.0	0.0	MEL
78	HIS	0.0	0.0	0.0	2.0	0.0	**98.0**	0.0	HIS
81	HIS	0.0	0.0	0.0	1.0	0.0	**98.5**	0.5	HIS
85	HIS	0.0	0.0	0.5	0.5	0.0	**94.6**	4.4	HIS
88	HIS	0.0	0.0	0.5	0.0	0.0	**99.5**	0.0	HIS
93	PCT	0.0	0.0	0.5	7.8	29.9	0.5	**61.3**	PCT
98	PCT	0.0	2.0	0.5	0.0	0.0	0.0	**97.5**	PCT
101	PCT	0.0	0.0	0.0	0.0	0.0	0.0	**100.0**	PCT
104	PCT	0.0	0.0	0.5	0.5	0.0	0.0	**99.0**	PCT

For each case, the percentage of representative tiles assigned to each diagnostic class is shown; Top1 (bold font, grey background), Top2 (grey background if >10%), and Top3 (grey background if >10%) indicate the 3 most prevalent predicted classes by majority vote. CAR, carcinoma; HIS, histiocytoma; LYM, lymphoma; MCT, mast cell tumor; MEL, melanoma; PCT, plasma cell tumor; STS, soft tissue sarcoma.

### 3.5 Whole-slide–level diagnostic performance (attention-free whole slide inference)

This analysis was performed on 28 independent full WSIs of the test set (4 cases per diagnostic category). The slides contained a median of 9,358 non-background tiles (mean 13,159), indicating that each whole-slide decision aggregated thousands of non-curated independent tile-level predictions. Across all 28 slides, the Top1 prediction matched the true diagnosis in 26 cases, corresponding to an overall slide-level accuracy of 92.9%. When allowing the correct diagnosis to appear within the top three ranked classes (Top1–Top3), all 28 slides (100%) were correctly identified within the three most prevalent predicted categories.

Performance was consistent across most diagnostic classes. All four cases of CAR, HIS, LYM, MCT and STS were correctly classified at the slide level (Top1 accuracy 4/4 for each class). MEL and PCT each had three correctly classified slides (Top1 accuracy 3/4), with a single misclassified case per class. In the misclassified MEL slide, MCT was the predominant predicted class (62.9% of non-background tiles) and MEL was ranked second (32.3% of tiles). In the misclassified PCT slide, STS was Top1 (34.5% of tiles), while PCT and MEL were close behind (23.4 and 24.8% of tiles, respectively); in both cases, the true diagnosis still appeared within the Top3 ranking.

On average, the Top1 class accounted for 73.4% of non-background tiles per slide (median 80.5%; range 34.5–97.3%), indicating that the majority-vote decision was typically supported by a large proportion of the slide, although some cases showed substantial mixture of competing classes. In 13/28 cases, the Top2 or Top3 classes were each represented by <10% of tiles, indicating a single strong diagnosis without differentials. In the remaining 15/28 cases, 2/4 MCT, 1/4 LYM, 4/4 CAR, 3/4 STS, 2/4 MEL, 1/4 HIS, and 2/4 PCT cases had plausible Top2 and/or Top3 differential diagnoses represented by >10% of tiles.

At the class level, the median fraction of tiles assigned to the correct Top1 diagnosis per slide ranged from approximately 48–86%, being lowest for CAR (median 48.6%) and highest for LYM (median 91.9%), with intermediate values for HIS, MCT, MEL, PCT and STS. Across the 28 whole slides, the mean Top1–Top2 margin was 59.4 percentage points, the median was 69.3, and the range was 9.7–95.4. No slides had margins below 5 percentage points. Two slides (2/28; 7.1%) had margins below 15 percentage points: Case 43 (10.3) and Case 93 (9.7). A visual representation of samples of WSI-level masks is presented in Figure 3 while a summary of the results is presented in Table 2.

**Figure 3:**
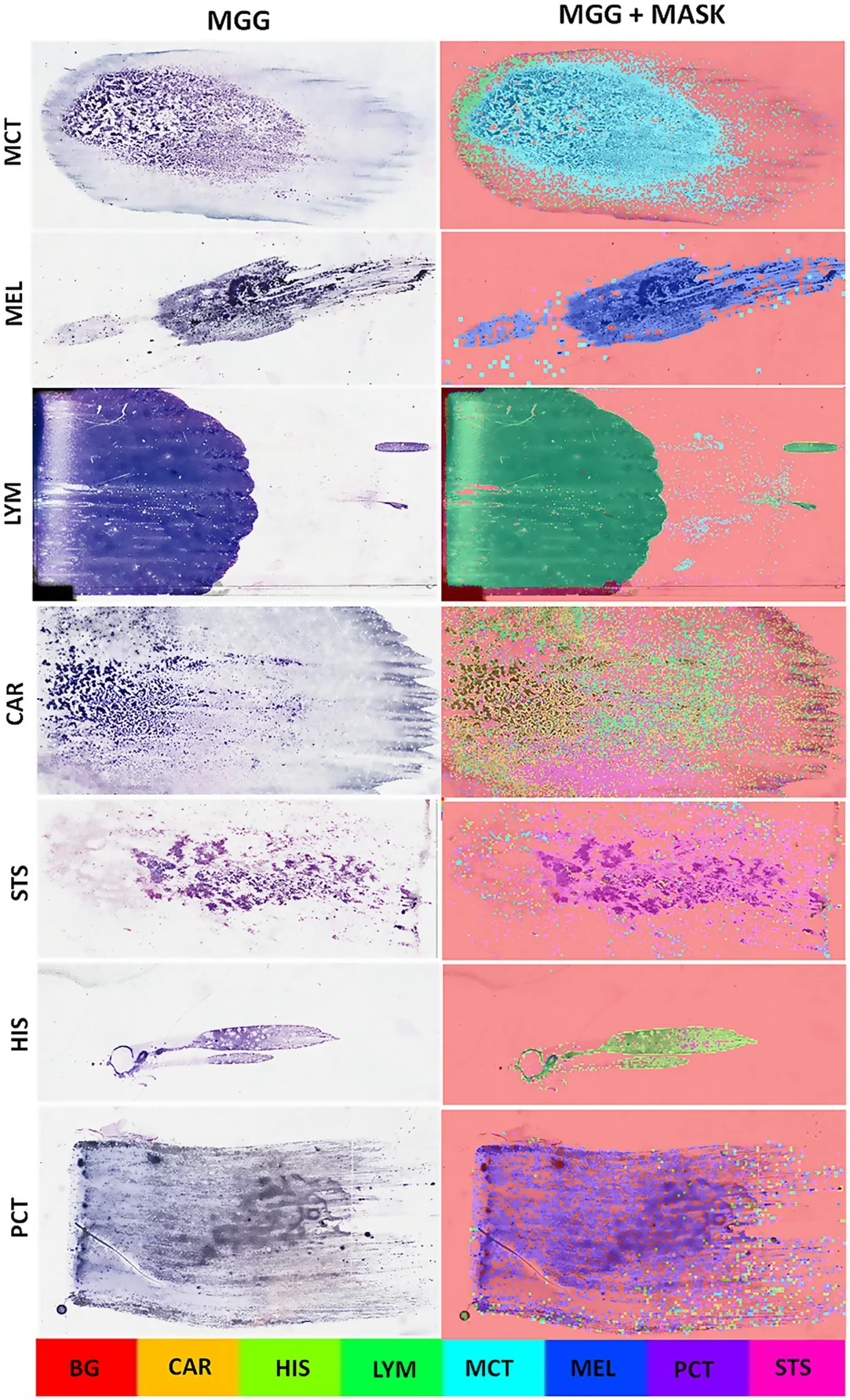
Representative whole-slide image (WSI)–level predictions generated by the trained convolutional neural network for 7 diagnostic categories. For each case, the original May–Grünwald Giemsa (MGG)–stained cytology WSI is shown on the left and the corresponding reconstructed classification mask on the right with pseudocolors indicating the predicted class: background (BG), carcinoma (CAR), histiocytoma (HIS), lymphoma (LYM), mast cell tumor (MCT), melanoma (MEL), plasma cell tumor (PCT), and soft tissue sarcoma (STS). Representative examples are shown for mast cell tumor, melanoma, lymphoma, carcinoma, soft tissue sarcoma, histiocytoma, and plasma cell tumor. In most cases, the predominant predicted class corresponded to the final slide-level diagnosis, while smaller proportions of tiles were assigned to other classes or to background, reflecting intralesional heterogeneity and the presence of non-diagnostic areas.

**Table 2:** Whole slide level diagnostic predictions obtained by attention-free whole slide inference.

Whole-slide–level diagnostic performance (*Attention-free whole slide inference*)									
Case	Ground truth	Predicted class (% non-background tiles)							Final diagnosis
		MCT	LYM	CAR	STS	MEL	HIS	PCT	
4	MCT	**97.3**	0.0	0.4	0.3	2.0	0.1	0.1	MCT
8	MCT	**43.6**	0.6	15.5	9.6	25.6	1.1	4.1	MCT
11	MCT	**86.8**	8.7	1.2	1.4	0.4	0.0	1.4	MCT
14	MCT	**61.9**	0.2	0.9	3.8	31.4	0.5	1.4	MCT
19	LYM	2.9	**91.0**	1.6	2.8	0.2	1.5	0.1	LYM
23	LYM	0.1	**92.8**	3.6	0.7	0.2	2.6	0.1	LYM
26	LYM	1.4	**94.3**	1.6	0.8	0.6	1.2	0.1	LYM
29	LYM	3.9	**66.5**	6.4	13.6	0.7	8.7	0.1	LYM
33	CAR	0.3	22.7	**48.8**	26.3	0.4	0.2	1.4	CAR
37	CAR	0.1	13.4	**50.9**	23.5	0.4	3.8	8.0	CAR
40	CAR	14.8	0.2	**48.5**	15.8	14.9	2.5	3.4	CAR
43	CAR	5.5	34.9	**45.2**	10.6	0.4	1.9	1.5	CAR
48	STS	2.6	7.0	14.3	**48.7**	4.2	14.7	8.3	STS
51	STS	0.3	0.8	11.8	**82.5**	0.4	1.2	3.1	STS
55	STS	0.1	0.1	10.9	**84.9**	1.8	0.6	1.7	STS
59	STS	3.2	1.4	3.5	**89.1**	0.0	1.9	0.8	STS
63	MEL	1.9	0.0	0.0	0.8	**97.3**	0.0	0.0	MEL
66	MEL	4.8	0.0	0.0	1.4	**93.8**	0.0	0.0	MEL
70	MEL	6.3	0.0	0.2	19.1	**74.3**	0.1	0.1	MEL
73	MEL	**62.9**	0.0	0.0	4.7	32.3	0.0	0.0	MCT
78	HIS	0.3	3.0	1.3	9.8	0.1	**85.3**	0.3	HIS
81	HIS	1.7	2.5	2.3	25.5	0.2	**65.6**	2.3	HIS
85	HIS	0.1	0.6	1.1	7.7	0.0	**85.4**	5.1	HIS
88	HIS	0.3	0.1	4.9	5.9	0.0	**88.0**	0.9	HIS
93	PCT	7.7	0.1	8.0	**34.5**	24.8	1.6	23.4	STS
98	PCT	0.0	13.1	3.4	2.0	0.0	0.5	**80.9**	PCT
101	PCT	2.3	0.5	4.0	9.3	0.2	3.6	**80.2**	PCT
104	PCT	2.9	0.3	5.5	9.4	2.7	3.7	**75.6**	PCT

For each case, the percentage of representative tiles assigned to each diagnostic class is shown; Top1 (bold font, grey background), Top2 (grey background if >10%), and Top3 (grey background if >10%) indicate the 3 most prevalent predicted classes by majority vote. CAR, carcinoma; HIS, histiocytoma; LYM, lymphoma; MCT, mast cell tumor; MEL, melanoma; PCT, plasma cell tumor; STS, soft tissue sarcoma.

## 4 Discussion

In this study, we demonstrated that a relatively simple deep CNN (Inception-v3) can achieve relative high accuracy in classifying seven typical examples of common canine tumor types from cytology whole-slide images (WSIs). This approach represents, to our knowledge, the first CNN based multi-tumor AI classification in veterinary cytology. The dataset included a balanced selection of these frequent cutaneous neoplasms, which represent a substantial proportion of canine skin tumors (20). Importantly, our dataset consisted of very typical and fully diagnostic tumor cases selected to establish a proof-of-concept performance on well differentiated cases. As a result, the reported accuracy likely overestimates performance in routine clinical cytology, where slides may have suboptimal cell preservation, mixed inflammatory and neoplastic populations, or neoplastic cells that are not well-differentiated. Future work must explicitly evaluate the model, or improvements of the same, on diagnostically more challenging cases to determine its true clinical utility.

In this work we used a rigorous case-wise split to ensure that no images from the same patient appeared in both training and testing sets, preventing data leakage. This practice follows recommended guidelines in computational pathology for avoiding data leakage and reflects real-world deployment, where models must generalize to new cases (21). Although our total sample size was modest by deep learning standards in human medicine studies, it is comparable to recent proof-of-concept studies in veterinary pathology that used ~50 images per class (22). Such dataset sizes can still yield relatively robust models when combined with careful augmentation and transfer learning, but they also underscore the need for larger multi-institutional datasets in the future to further improve generalizability (23, 24). We selected Inception-v3 because, for many histopathology problems, a well-regularized mid-complexity CNN can generalize as well as, or better than, more complex networks. When labeled datasets are modest and discriminative cues are dominated by multi-scale texture and cytomorphology, very high-capacity models can overfit to slide-specific artifacts, whereas smaller backbones are constrained to learn more transferable features (25, 26).

Our CNN achieved strong performance at the tile level, correctly classifying most cytological fields of view. Notably, the model’s patch-level precision (~85%) was comparable to reports in similar histopathology tasks (22). This level of accuracy is remarkable given the unique challenges of cytology versus histology such as the lack of meaningful tissue structure and differences in smear staining, cell preservation, and sampling technique, making cytology a more difficult domain for image analysis in general. One veterinary study reported cytology sensitivities of approximately ~69% for differentiating malignant from benign soft-tissue tumors (27).

The three CNNs fitted training tiles almost perfectly (mean training accuracy 0.9993), while mean test accuracy across folds was 0.8630 (range 0.793–0.901) with high macro balanced accuracy (0.9201). This generalization gap is consistent with patch-based workflows where inter-case biological and technical variability can exceed within-case variability. Crucially, patch-based pipelines can overestimate performance if tiles from the same case leak into both training and testing partitions; therefore, rigorous case-wise splitting is essential for credible performance reporting and realistic clinical translation (28). Performance remained robust for LYM/MCT/MEL/CAR, while PCT/HIS/STS/BG were more variable, and confusion matrices showed most misclassifications between PCT-HIS-STS and STS–CAR. This pattern suggests that errors are primarily driven by morphologic overlap, rather than broad network instability.

However, recent medical-imaging studies, applied to different disciplines than cytology, propose strategies that could complement this proof-of-concept baseline. Uyar and colleagues selected the three strongest of 15 pretrained CNNs and combined them using Artificial Bee Colony-optimized weighted voting for eye-disease classification, increasing accuracy from 93.17% for the strongest single model to 98.84% for the ensemble, which achieved an F1-score of 98.85% (29). Such diversity could reduce model-specific variance and stabilize ambiguous cytology predictions. Yurdakul and Taşdemir also investigated an ensemble of CNNs and a Vision Transformer for brain-tumor detection (30). This local–global combination is attractive for cytology because convolutions can emphasize cellular morphology while self-attention may capture relationships among spatially separated image regions. However, ensembles increase training, inference, and calibration costs, while transformer-based models generally require substantial data or strong pretraining; with only 91 independent cases, flexible architecture or weight selection could itself overfit unless performed within nested case-wise validation and followed by external testing.

Yurdakul and Taşdemir subsequently integrated EfficientNetV2 with an attention-based MLP-Mixer and Grad-CAM, reporting 98.53% accuracy under stratified five-fold cross-validation on 3,064 brain MRI images (31). This supports evaluation of efficient hybrids that combine multi-scale features, token mixing, attention, and explainability. Nevertheless, MRI and cytology WSIs have different image structure and sources of domain shift, and image-level performance cannot be assumed to transfer to case-wise or cross-institutional cytology. Yurdakul et al. combined a Swin-Mamba U-Net with region-wise Bézier intensity augmentation for domain-generalized brain-tumor segmentation and observed a larger gain on external target datasets than on the source dataset (32). The principle of training with biologically plausible intensity perturbations is relevant to staining and scanner variation in cytology, but their label-aware, mask-based augmentation addresses segmentation rather than multiclass WSI classification.

Accordingly, in our study inception-v3 was selected as a starting point, given the lack of AI- multi class tumor classification approaches in veterinary cytology, as a well-established ImageNet-pretrained baseline that was compatible with the available MATLAB workflow and modest case count. Ensembles, transformer hybrids, attention modules, and state-space models may improve robustness, but architectural complexity alone cannot eliminate the observed gap between near-perfect training accuracy (99.93%) and mean test accuracy (86.30%), which also reflects limited inter-case and inter-institutional diversity. Future studies should test nested validation for architecture and ensemble-weight selection, confidence calibration with uncertainty-aware decision thresholds, and independent multi-institutional cohorts.

To further understand the clues used by the network to make a decision, we also explored model interpretability via Grad-CAM visualization, which provided insights into what cytologic features the CNN relied upon. The Grad-CAM heatmaps consistently highlighted the areas of slides containing clusters or single intact neoplastic cells, while ignoring background artifacts such as excessive blood or slide debris. In many instances, the CNN’s attention maps corresponded to the same cellular features that a cytologist would consider diagnostic, which is very encouraging. We believe that this form of explainable AI is crucial for building trust in co-pilots or automated diagnostic systems and Grad-CAM-style visualization supports qualitative validation of model attention (33).

To minimize selection bias and preserve an independent evaluation, we carried forward the intermediate/average-performing Network 3 for Grad-CAM and all subsequent ROI- and whole-slide analyses. Although Network 1 achieved the highest tile-level test accuracy, selecting the best-performing fold post-hoc may yield an overly optimistic estimate; such reuse of held-out performance for model choice is a recognized source of selection bias in performance evaluation (28). Crucially, Network 3’s ROI- and WSI-level experiments were conducted only on its corresponding held-out test fold, which did not influence training and was separated case-wise as previously discussed. This choice has direct implications for aggregation: majority-vote ROI and WSI results derived from Network 3 can be interpreted as end-to-end performance of the full pipeline on previously unseen cases under “typical” tile-level behavior, rather than a best-case network. We acknowledge however that limitations remain: our analyses were demonstrated on a single laboratory dataset fold and future work should include retraining on all folds and validation in external, preferably prospective, cohorts (e.g., different laboratories). However, the development of locally-trained/deployed, quality-controlled networks for single lab use may represent the future of the use of CNNs compared to the promise of large cloud models aiming at solving the impossible problem of “global generalizability” (34).

At ROI level, majority vote across 204 human-selected representative tiles per case produced 100% Top1 diagnosis accuracy across 28 cases. In most ROIs (21/28), Top2 and Top3 classes each represented <10% of tiles, indicating that the decision was usually supported by a highly dominant class after curation. This aligns with evidence that expert viewing behavior concentrates on diagnostically salient regions, offering a usable “attention prior” that reduces background noise at inference (35). Although the correct class ranked first in all 28 curated ROIs, two predictions were near-ties. The Top1–Top2 analysis highlighted this variation: despite a median margin of 95.8 percentage points, Cases 48 and 73 had margins of only 1.0 and 1.4 percentage points, respectively, indicating little separation between the two leading classes. This margin describes the balance of tile-level class assignments within an ROI, rather than a calibrated probability that the diagnosis is correct. Thus, the 100% Top1 accuracy should not be interpreted as guaranteeing correct whole-slide classification, as illustrated by the subsequent misclassification of Case 73. These cases show that rank-based accuracy should be accompanied by class proportions, margins, and ultimately calibrated uncertainty or an abstention threshold before clinical deployment.

In attention-free whole-slide inference, each decision aggregated thousands of non-background tiles (median 9,358; mean 13,159) and achieved 92.9% Top1 accuracy (26/28) with 100% Top3 agreement. Notably, slide-level accuracy exceeded mean single-tile accuracy, consistent with majority vote suppressing sporadic tile-level errors. Compared with the curated ROIs, whole-slide inference showed less class separation, with a lower median Top1–Top2 margin (69.3 versus 95.8 percentage points). Only two whole-slide predictions had margins below 15 percentage points: Case 43 was correctly classified, whereas Case 93 was misclassified; conversely, the other misclassified slide, Case 73, retained a margin of 30.6 percentage points. Thus, the Top1–Top2 margin reflects the relative dominance of the leading predicted class but should not be interpreted as a calibrated indicator of diagnostic correctness. Majority vote is a recognized baseline WSI aggregation method; however, the observed class-mixture highlights why more adaptive aggregation (e.g., attention-based pooling) is increasingly used for slide-level inference (21).

The two majority vote inference modes (*attention-guided inference* and *attention-free whole slide inference*) map to different clinical roles. Attention-guided inference simulates an AI “co-pilot”: the clinician chooses representative ROIs and the model provides a rapid, standardized second opinion on material already judged relevant. Its strengths are high decisiveness, shorter time, lower computational burden and clearer attribution of errors to tile content; its limitations are operator dependence, potential sampling bias, and the possibility of missing focal components outside the selected ROI. Attention-free whole-slide inference is a stricter test of robustness and better reflects autonomous screening/triage; its strengths are comprehensive coverage and spatial prediction mapping, while its limitations include sensitivity to background prevalence and class mixture, slower speed, and higher compute (21).

Despite the promising results, our study has limitations that warrant discussion. The dataset size remains a weakness, which is typical of veterinary studies. Our training set may not capture the full morphologic diversity present even within each tumor type. Expanding the dataset with additional cases, especially from different institutions, is a clear next step. Lack of such external validation is a common weakness in many inaugural AI studies, and we acknowledge that our results represent performance under some controlled conditions.

The case-selection criteria impose an additional limitation. Because poorly differentiated tumors and generally poor-quality preparations were excluded, the reported estimates are conditional on slides with typical morphology and adequate diagnostic material. The present cohort cannot quantify the magnitude of performance loss in unselected practice. Qualitatively, lower cellularity, poor preservation, blood or inflammatory contamination, mixed neoplastic populations, and loss of lineage-specific morphology are expected to reduce Top1 dominance and increase confusion among overlapping classes. A clinically representative validation should therefore enroll consecutive cases, retain indeterminate and non-diagnostic samples, pre-specify specimen-quality and diagnostic-difficulty strata, and report accuracy, calibration, and abstention/referral rates within each stratum and across external laboratories. Until such testing is completed, these results should not be extrapolated to diagnostically ambiguous routine submissions.

Another limitation is the scope of tumor classes addressed. We focused on seven well-defined tumor types; real-world cytology practice, however, must also contend with samples including less common neoplasms. Performance in poorly differentiated, low-cellularity, or otherwise diagnostically difficult routine samples cannot be inferred from the present curated cohort.

To our knowledge, no prior study has attempted multi-class CNN classification across a spectrum of different tumor types in veterinary cytology WSIs. This represents an encouraging step towards the development of AI assisted cytological diagnosis, although a larger, prospective and external dataset should be tested with different architectures to confirm the clinical applicability of our approach.

## Data Availability

The curated dataset and MATLAB codes used for this work are available at: Zenodo.org; DOI 10.5281/zenodo.19493409.
